# Adropin Alleviates Myocardial Fibrosis in Diabetic Cardiomyopathy Rats: A Preliminary Study

**DOI:** 10.3389/fcvm.2021.688586

**Published:** 2021-07-12

**Authors:** Mao Liu, Jiao Ai, Zhuang Shuai, Kai Tang, Zongyu Li, Yin Huang

**Affiliations:** ^1^Department of Cardiology, Cardiovascular Research Center, Affiliated Hospital of North Sichuan Medical College, Nanchong, China; ^2^Department of Rheumatology, Affiliated Hospital of North Sichuan Medical College, Nanchong, China; ^3^Department of Cardiology, Suining Central Hospital, Suining, China; ^4^Department of Cardiology, The Fifth Affiliated Hospital of Sun Yat-sen University, Zhuhai, China

**Keywords:** myocardial fibrosis, adropin, mitochondrial dynamics, mitofusin, diabetic cardiomyopathy

## Abstract

**Aim:** Adropin (ADR) is a novel regulatory polypeptide and has important effects on energy metabolism in the heart. However, it is still unclear whether ADR can relieve ventricular remodeling in DCM. Therefore, this study was conducted to assess the effect of ADR on myocardial fibrosis in DCM rats.

**Materials and Methods:** Twenty Wistar rats were randomly assigned into four groups: healthy control group (CON), DCM model group (DCM), DCM model treated with ADR group (ADR) and DCM model treated with perindopril group (PER). Collagen volume fraction (CVF) and perivascular collagen area (PVCA) were calculated. Diastolic function was assessed by echocardiography. The mitochondrial membrane potential assay was conducted by Rhodamine 123 staining. The protein expression levels of Col I, Col III, Mitofusin-1, Mitofusin-2 and Drp1 were evaluated using western blot.

**Results:** Compared to CON group, CVF, PVCA and the relative protein expression of Col I, Col III and Drp1 increased in DCM group. And the relative expression of Mitofusin-1 and Mitofusin-2 proteins decreased. During our investigations, CVF, PVCA and the relative protein expression of Col I, Col III and Drp1 decreased in ADR treated rats compared to DCM group. The diastolic function was elevated in ADR group. The fluorescence of Rhodamine 123 and the expression of Mitofusin-1 and Mitofusin-2 also increased in ADR group.

**Conclusion:** Our study demonstrated that ADR could alleviate myocardial fibrosis and improve diastolic function in DCM rats. ADR may be a putative candidate for the treatment of DCM.

## Introduction

Diabetic cardiomyopathy (DCM) is a type of cardiovascular damage and recognized as a specific entity in patients with type 2 diabetes mellitus, excluding coronary atherosclerotic heart disease, hypertrophic cardiomyopathy, hypertensive heart disease and other heart diseases (1, 2). Metabolic disturbances, myocardial fibrosis, small vessel disease and autonomic dysfunction are associated with the development of DCM. The number of deaths caused by diabetes mellitus and its complications is as high as 1.5 to 5 million each year. In addition, more than half of diabetes mellitus related fatal or disabling events are caused by DCM (3).

Mitochondria play a pivotal role in cellular energy transduction. One-third of the heart's volume is composed of mitochondria alone as they are essential organelles in the production of adenosine triphosphate from the oxidation of fatty acid and glucose (4). Mitochondrial dysfunction may contribute to the development of DCM by inducing insulin resistance in skeletal muscle, adipose tissue and pancreatic β-cells (5).

Recent studies have found that Adropin (ADR) is closely related to cardiovascular disease (6–8). ADR, a regulatory polypeptide involved in energy homeostasis, is composed of 76 amino acids and encoded by genes related to energy balance. It is mainly expressed in tissues such as the heart, liver and brain. Besides, it is also expressed in coronary arteries and umbilical veins (9, 10).

ADR can regulate lipid metabolism and insulin resistance. ADR-treated mouse hearts showed improved cardiac efficiency and enhanced insulin signaling compared to control hearts. ADR-treatment protocols *in vivo* and *ex vivo* induced a reduction in the inhibitory phosphorylation of pyruvate dehydrogenase and the protein levels of the responsible kinase pyruvate dehydrogenase kinase 4. These results indicated that ADR has important effects on energy metabolism in the heart (11). ADR can also reduce the risk of atherosclerosis and myocardial ischemia/reperfusion injury by activating the RISK signal transduction pathway (12, 13). Therefore, ADR may be a putative candidate for the treatment of cardiac disease associated with impaired insulin sensitivity (7, 11, 14).

However, it is still unclear whether ADR can relieve ventricular remodeling by regulating mitochondrial dynamics in DCM. Therefore, the present study was conducted to assess the effect of ADR myocardial fibrosis in DCM rats.

## Materials and Methods

### Subjects

Twenty Wistar rats were purchased from Model Animal Research Center of Nanjing University (SCXK Su. 2015-0001). All rats were 8 weeks of age at the beginning of the study. The rats were housed individually under room temperatures with 12: 12 h light/dark cycle and free access to food and water for the duration of the study. All experimental procedures were approved by Ethics Committee of North Sichuan Medical College and complied with the guidelines for the Care and Use of Laboratory Animals.

### Experimental Design

The rats were randomly assigned into four groups: healthy control group (CON), DCM model group (DCM), DCM model treated with ADR group (ADR) and DCM model treated with perindopril group (PER). In CON group, rats were given normal saline gavage (1 ml/100 g/d) for 4 weeks without modeling operation. In DCM group, rats were given normal saline gavage (1 ml/100 g/d) for 4 weeks after modeling operation. In ADR group, rats were given ADR gavage (5 μg/100 g/d) for 4 weeks after modeling operation (15). In PER group, rats were given PER gavage (200 μg/100 g/d) for 4 weeks after modeling operation (16). After the intervention, all rats were sacrificed with cervical dislocation.

### DCM Rat Modeling

One-time intraperitoneal injection of 1% streptozotocin at a dose of 70 mg/kg was used to destroy the function of pancreatic β cells. The rats' tail vein blood samples were taken on the third and seven day after the injection to test the fasting blood glucose. If both the blood glucose were more than 16.7 mmol/L, and the rats had polydipsia, polyphagia and polyuria, a rat model of DCM was established (17, 18).

### Echocardiography

Two-dimensional and Doppler echocardiography was performed to assess diastolic function using a GE Vivid 7 echocardiograph with a 10-MHz phased array probe. Ratio of peak velocity of early (E) to late atrial (A) mitral flow (E/A) was detected. Circumferential Strain rate in early (SRe) and late (SRa) diastole were obtained from parasternal short-axis gray-scale images.

### Left Ventricular Weight/Body Weight Ratio

Body weights were measured before the rats were sacrificed. The left ventricle was freed immediately after the heart was taken. An electronic balance was used to weigh the left ventricular mass. LVW/BW ratio (mg/g) = left ventricular weight (mg)/body weight (g) (19).

### Collagen Volume Fraction and Perivascular Collagen Area

Masson staining was used to evaluate the severity of myocardial fibrosis (20). Based on Masson staining sections of myocardial tissues, CVF and PVCA were calculated by Image Pro Plus software version 6.0. Masson tri-color staining kit were purchased from KeyGEN Biotech (Nanjing, China).

### Immunohistochemistry of Collagen I

Histological determinations of cardiac fibrosis were performed in 5 μm thick sections of paraffin-embedded myocardial tissue. The immunochemistry was performed following the protocol. For each immunochemistry and staining, serial sections were done. The most representative image was shown. Image J software was used to quantitatively calculate the expression of Col I in myocardial tissue.

### Rhodamine 123

The mitochondrial membrane potential assay in myocardial tissue was conducted by Rhodamine 123 staining. Disruption of mitochondrial potential has been shown to be featured with the decrease in Rhodamine 123 retention and fluorescence (21). The microtubes containing 1 mL of mitochondrial solution mixed with 10 μl of rhodamine 123 dye solution were incubate at 37°C for 1 h in the dark. After washing three times with PBS, the fluorescence intensity was measured with a microplate reader.

### Western Blot

The protein expression levels of Col I and Col III in myocardial tissue were evaluated using western blot, with glyceraldehyde-3-phosphate dehydrogenase (GAPDH) protein as the reference. The relative proteins of mitochondrial dynamics such as Mitofusin-1, Mitofusin-2 and Drp1 in myocardial tissue were also detected using western blot, with voltage-dependent anion channel (VDAC) protein as the reference. About 100 mg of myocardial tissue were cut into small pieces and put into a pre-cooled EP tube. Total protein concentration was determined according to the instructions of the BCA kit. The supernatants were collected. Then they were separated by SDS-polyacrylamide gels and transferred to a PVDF membrane. The membranes were blocked with 5% skimmed milk powder TBST solution for 1 h at room temperature and incubated with primary antibodies overnight at 4°C. The remaining liquid on the membrane was washed with TBST. Images were captured with Tanon 6600 luminescence imaging workstation (Tanon Science & Technology Co., Ltd. Shanghai, China).

### Statistical Analysis

Data were presented as group mean and its standard error (mean ± SEM). One-way analysis of variance (ANOVA) followed by the least significant difference (LSD) *post hoc* test was used for the statistical analysis of the difference between multiple groups. All statistical analyses were performed in SPSS 20.0 (IBM Corp., Armonk, USA).

## Results

### Effects of ADR on Diastolic Function and LVW/BW Ratio

**Figure 1:**
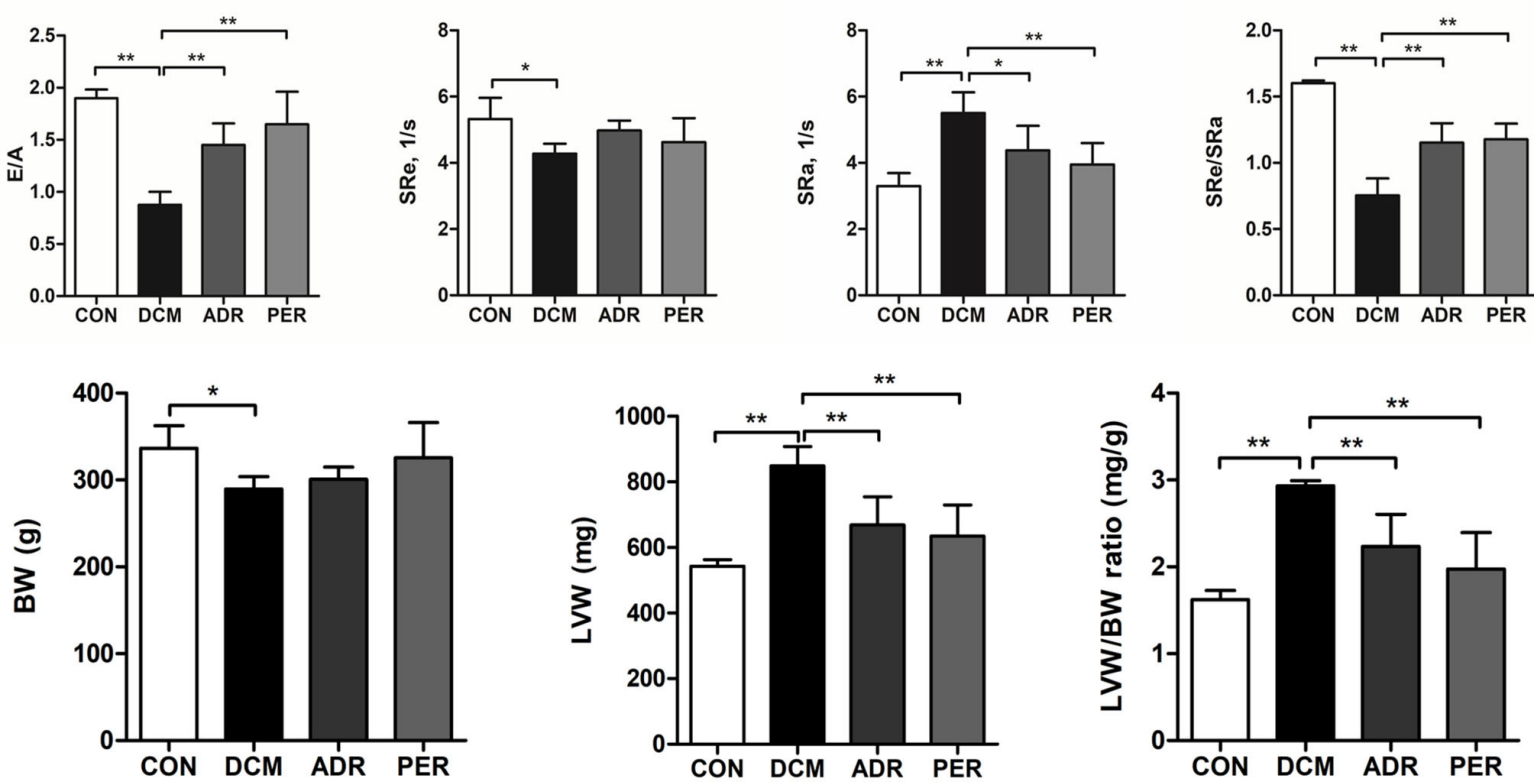
Effects of ADR on diastolic function and LVW/BW ratio in DCM rats. E/A ratio and SRe/SRa ratio dercreased in DCM group compared with CON group. Compared to DCM group, E/A ratio and SRe/SRa ratio increased significantly in ADR group. Compared to CON group, LVW/BW ratio increased in DCM group. ADR can decrease LVW/BW ratio significantly. It also decreased in PER group, compared with DCM group. CON, control; DCM, diabetic cardiomyopathy; ADR, adropin; PER, perindopril; E/A, ratio of peak velocity of early (E) to late atrial (A) mitral flow. SRe and SRa, strain rate in early and late diastole, respectively. LVW, left ventricular weight; BW, body weight. Values are expressed as mean ± standard deviation. *P < 0.05, **P < 0.001.

As shown in Figure 1, E/A ratio and SRe/SRa ratio were used to assess the diastolic function of DCM rats. LVW/BW ratio was used to assess the severity of cardiac remodeling in DCM rats. E/A ratio (1.90 ± 0.09 vs. 0.89 ± 0.12, *P* < 0.001) and SRe/SRa ratio (1.60 ± 0.02 vs. 0.75 ± 0.13, *P* < 0.001) dercreased in DCM group compared with CON group. Compared to DCM group, E/A ratio (1.43 ± 0.18 vs. 0.89 ± 0.12, *P* = 0.001) and SRe/SRa ratio (1.15 ± 0.15 vs. 0.75 ± 0.13, *P* < 0.001) increased significantly in ADR group. LVW (543.26 ± 19.44 mg vs. 849.05 ± 58.91 mg, *P* < 0.001) and LVW/BW ratio (1.62 ± 0.11 mg/g vs. 2.93 ± 0.06 mg/g, *P* < 0.001) increased in DCM group compared with CON group. Compared to DCM group, LVW/BW ratio decreased significantly in ADR (2.23 ± 0.37 mg/g vs. 2.93 ± 0.06 mg/g, *P* = 0.005) and PER group (1.98 ± 0.42 mg/g vs. 2.93 ± 0.06 mg/g, *P* = 0.001).

### Effects of ADR on CVF and PVCA of Myocardial Tissue

**Figure 2:**
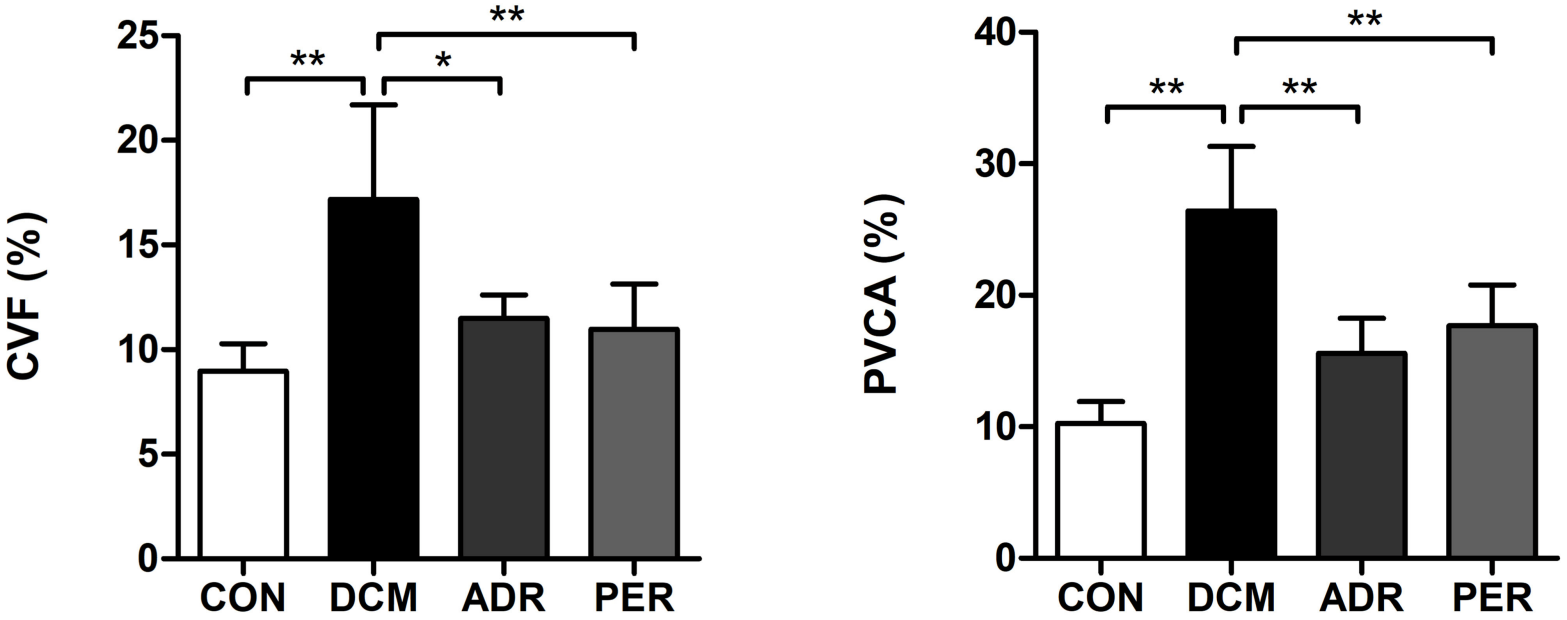
Effects of ADR on CVF and PVCA of myocardial tissue in DCM rats. Compared to DCM group, CVF and PVCA decreased in ADR and PER group. CON, control; DCM, diabetic cardiomyopathy; ADR, adropin; PER, perindopril; CVF, collagen volume fraction; PVCA, perivascular collagen volume area. Values are expressed as mean ± standard deviation. *P < 0.05, **P < 0.01.

Using Masson staining sections and Image Pro Plus version 6.0 software, CVF and PVCA of myocardial tissue were calculated (Figure 2). Compared to CON group, CVF (8.97 ± 1.31% vs. 17.18 ± 4.53%, *P* = 0.001) and PVCA (10.24 ± 1.68% vs. 26.41 ± 4.92%, *P* < 0.001) in DCM group increased dramatically. CVF and PVCA in ADR group (11.49 ± 1.12% vs. 17.18 ± 4.53%, *P* = 0.001; 15.58 ± 2.67% vs. 26.41 ± 4.92%, *P* < 0.001) and PER group (10.98 ± 2.16% vs. 17.18 ± 4.53%, *P* = 0.001; 17.67 ± 3.12% vs. 26.41± 4.92%, *P* < 0.001) decreased significantly in the comparison with DCM group.

### Effects of ADR on Immunohistochemistry of Col I in Myocardial Tissue

**Figure 3:**
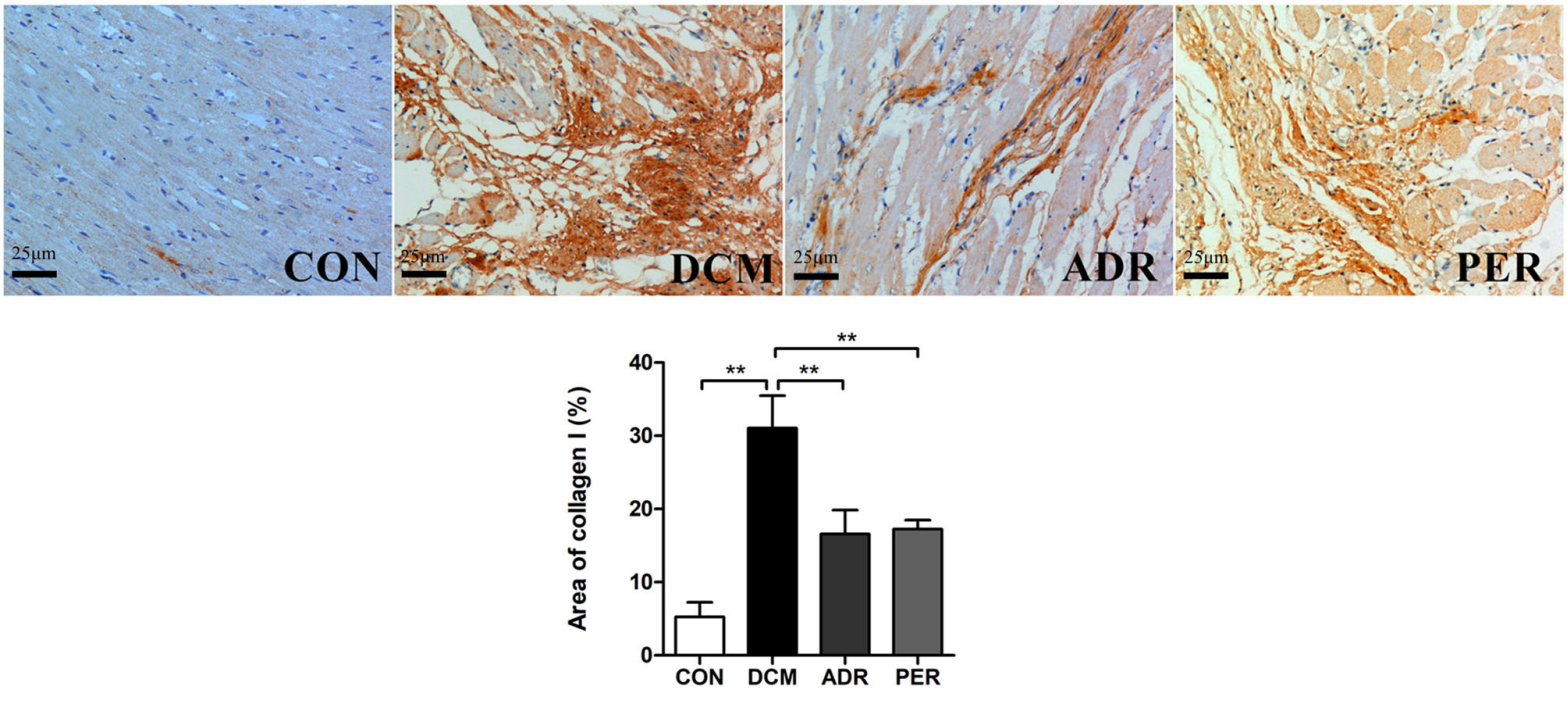
Immunohistochemistry of Col I in myocardial tissues in DCM rats (x 200). Compared to DCM group, the color of collagen fibers in ADR group and PER group becomes lighter and the expression of Col I reduced remarkably. CON, control; DCM, diabetic cardiomyopathy; ADR, adropin; PER, perindopril. Values are expressed as mean ± standard deviation. *P < 0.05, **P < 0.01.

The immunohistochemical staining of Col I in myocardial tissue was used to evaluate the severity of cardiac fibrosis (Figure 3). Collagen fibers from greenish yellow to orange red and red color as the severity of the cardiac fibrosis increased. In CON group, a small amount of Col I was scattered and the myocardial tissues arranged neatly. The Col I expression increased significantly in DCM group, showing strong positive staining in the form of flake. Quantitative calculation of the Col I expression in myocardial tissue was also made via Image J software. The Col I volume fractions in CON, DCM, ADR and PER group were 5.26 ± 1.98%, 31.06 ± 4.42%, 16.57 ± 3.29%, and 17.25 ± 1.24%, respectively. Compared with the DCM group, the color of collagen fibers in ADR group and PER group becomes lighter and the expression of Col I reduced remarkably (*P* < 0.001).

### Effects of ADR on Relative Expression of Col I and Col III Proteins

**Figure 4:**
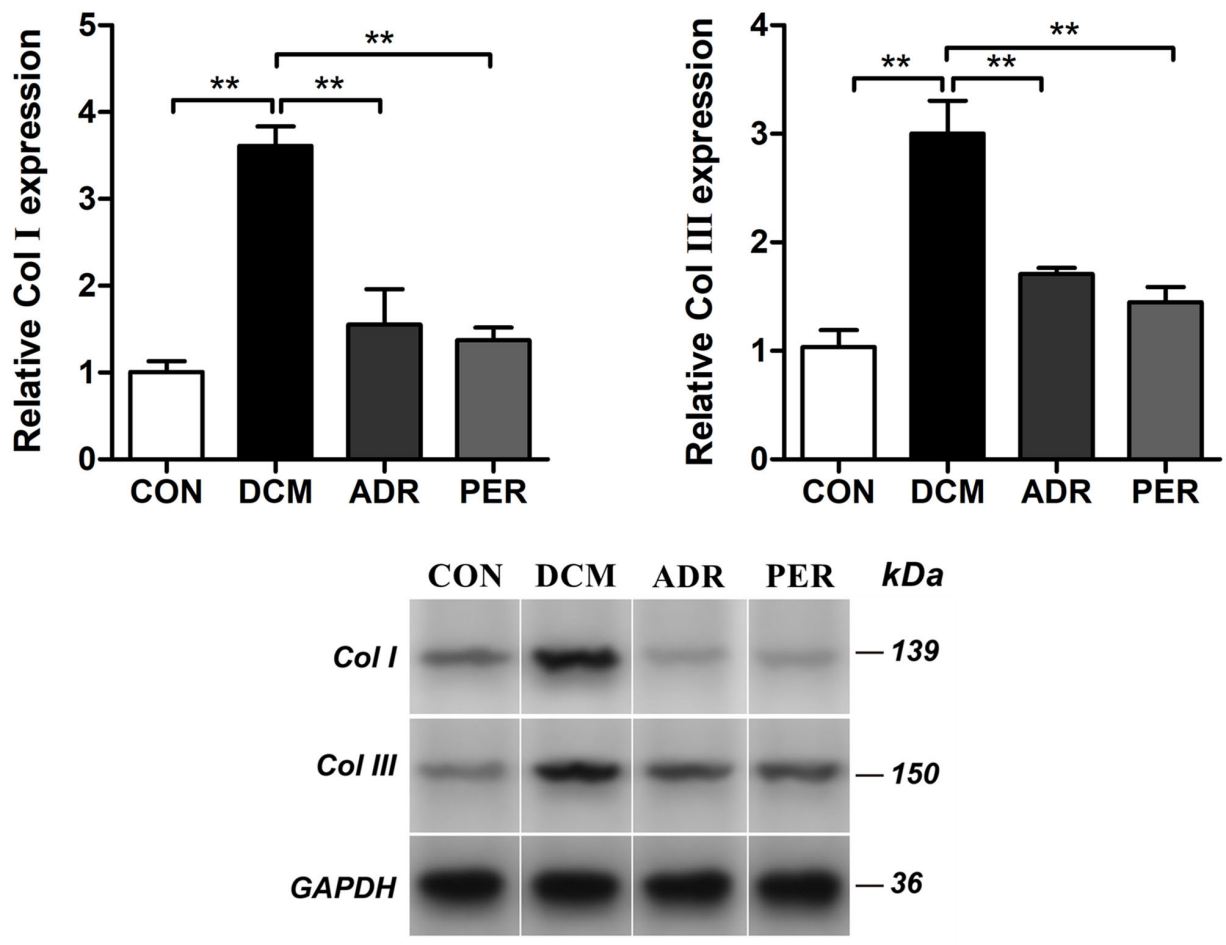
Effects of ADR on relative expression of Col I and Col III in myocardial tissue in DCM rats. Compared to DCM group, the relative expression of Col I and Col III proteins decreased in ADR and PER group. CON, control; DCM, diabetic cardiomyopathy; ADR, adropin; PER, perindopril; Col, collagen. Values are expressed as mean ± standard deviation. *P < 0.05, **P < 0.01.

The expression of Col I and Col III proteins in myocardial tissue were detected with Western Blot (Figure 4). Compared to CON group, the relative expression of Col I (1.01 ± 0.13 vs. 3.61 ± 0.23, *P* < 0.001) and Col III (1.03 ± 0.15 vs. 3.00 ± 0.30, *P* < 0.001) increased dramatically in DCM group. The relative expression of Col I decreased in ADR (1.55 ± 0.41 vs. 3.61 ± 0.23, *P* < 0.001) and PER (1.37 ± 0.15 vs. 3.61 ± 0.23, *P* < 0.001) group in the comparison with DCM group. Besides, compared to DCM group, the relative expression of Col III also reduced in ADR (1.71 ± 0.06 vs. 3.00 ± 0.30, *P* < 0.001) and PER (1.45 ± 0.14 vs. 3.00 ± 0.30, *P* < 0.001) group.

### Effects of ADR on the Fluorescence of Rhodamine 123

**Figure 5:**
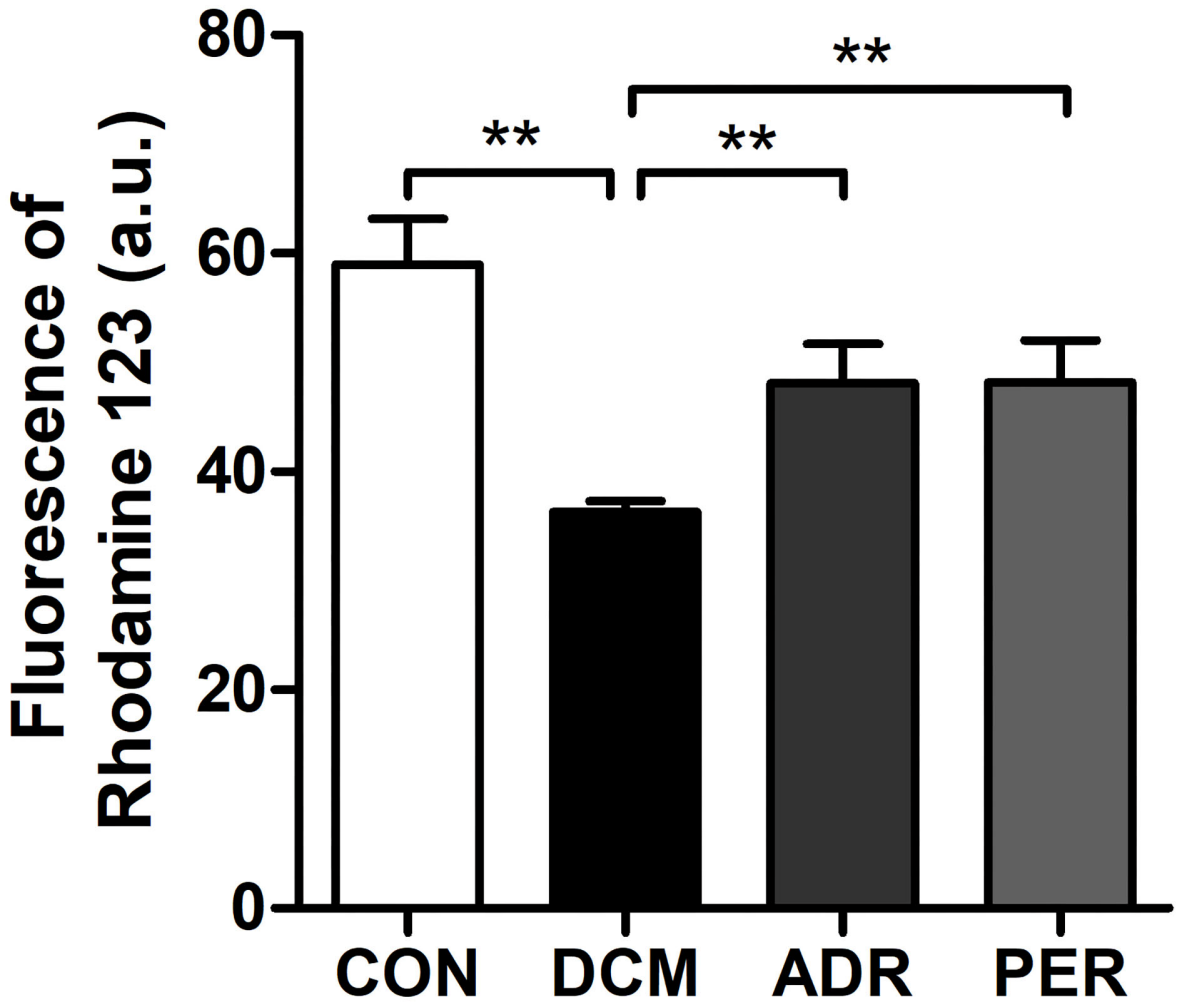
Effects of ADR on the fluorescence of Rhodamine 123 in DCM rats. Compared to DCM group, the fluorescence of Rhodamine 123 increased in ADR and PER group. CON, control; DCM, diabetic cardiomyopathy; ADR, adropin; PER, perindopril. Values are expressed as mean ± standard deviation. *P < 0.05, **P < 0.01.

Alteration in the mitochondrial transmembrane potential in myocardial tissue was determined by Rhodamine 123 staining. Disruption of the mitochondrial membrane followed by the loss in the mitochondrial membrane potential is a key step in the mitochondrial apoptotic pathway. As shown in Figure 5, the fluorescence of Rhodamine 123 in DCM group (58.97 ± 4.19 vs. 36.28 ± 1.04, *P* < 0.001) decreased significantly in the comparison with CON group. However, the fluorescence of Rhodamine 123 in ADR (48.10 ± 3.58 vs. 36.28 ± 1.04, *P* < 0.001) and PER group (48.19 ± 3.85 vs. 36.28 ± 1.04, *P* < 0.001) increased in the comparison with DCM group.

### Effects of ADR on the Relative Expression of Proteins in Mitochondrial Dynamics

**Figure 6:**
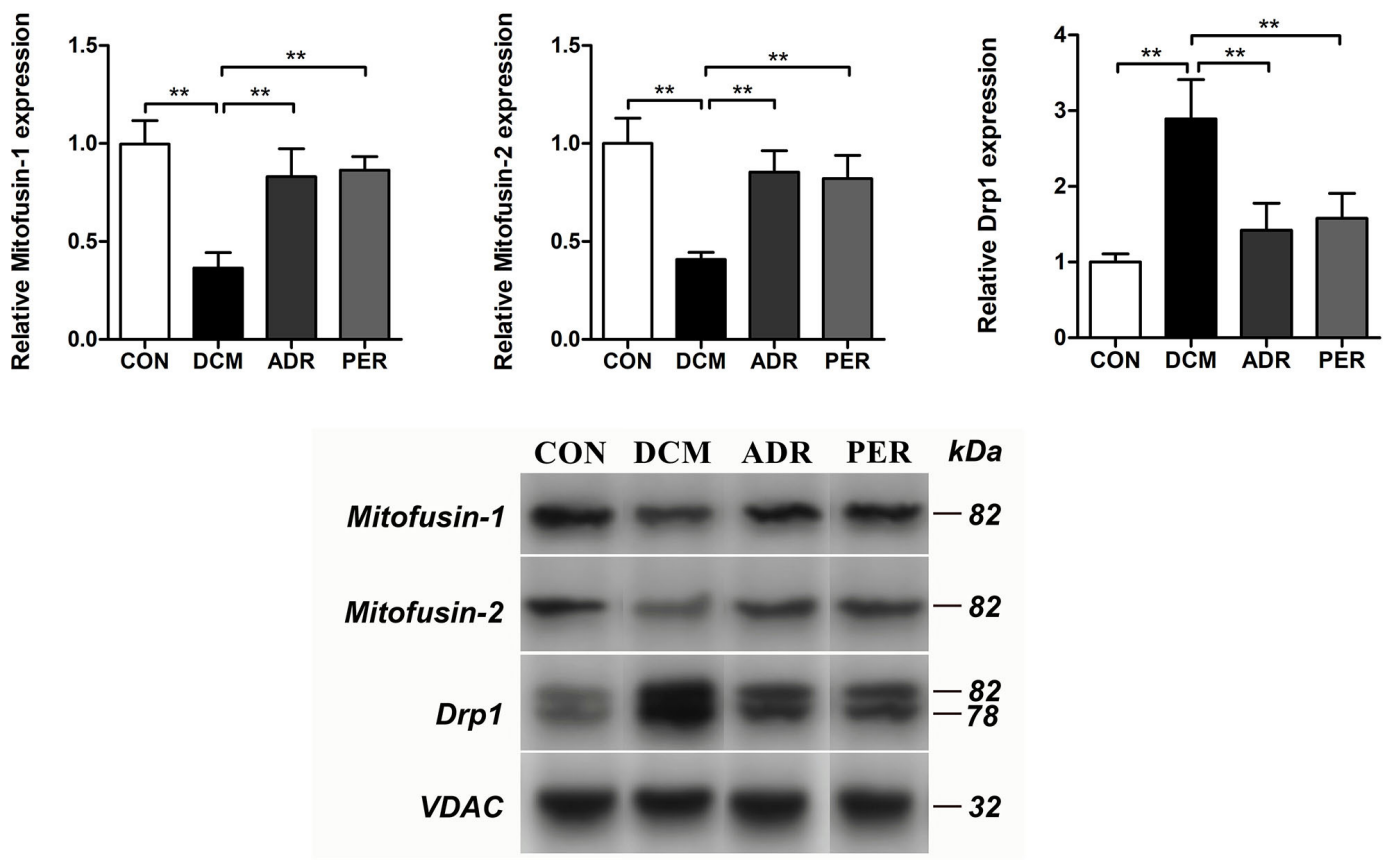
Effects of ADR on the relative proteins of mitochondrial dynamics in DCM rats. Compared to DCM group, the relative expression of Mitofusin-1 and Mitofusin-2 proteins increased in ADR and PER group. The relative expression of Drp1 protein decreased in ADR and PER group in comparison with DCM group. CON, control; DCM, diabetic cardiomyopathy; ADR, adropin; PER, perindopril. Values are expressed as mean ± standard deviation. *P < 0.05, **P < 0.01.

The relative proteins of mitochondrial dynamics in myocardial tissue were detected with Western Blot (Figure 6). Compared to CON group, the relative expression of Mitofusin-1 (1.00 ± 0.12 vs. 0.36 ± 0.08, *P* < 0.001) and Mitofusin-2 (1.00 ± 0.13 vs. 0.41 ± 0.04, *P* < 0.001) proteins decreased dramatically. The relative expression of Mitofusin-1 and Mitofusin-2 proteins increased in ADR (0.83 ± 0.14 vs. 0.36 ± 0.08, *P* = 0.001; 0.85 ± 0.11 vs. 0.41 ± 0.04, *P* = 0.001) and PER (0.86 ± 0.07 vs. 0.36 ± 0.08, *P* < 0.001; 0.82 ± 0.12 vs. 0.41 ± 0.04, *P* = 0.001) group, respectively, in comparison with DCM group. Compared to CON group, the relative expression of Drp1 protein (1.00 ± 0.11 vs. 2.89 ± 0.52, *P* < 0.001) increased significantly. However, it decreased in ADR (1.42 ± 0.36 vs. 2.89 ± 0.52, *P* = 0.001) or PER (1.58 ± 0.33 vs. 2.89 ± 0.52, *P* = 0.002) group in comparison with DCM group.

## Discussion

DCM is one of the most important complications of diabetes mellitus. Mitochondrial dysfunction is an important factor in the occurrence of DCM. The imbalance of mitochondrial dynamics is generally considered the cause of DCM. In the diabetic condition, total adenosine triphosphate was generated from fatty acid oxidation instead of glucose, which generates more reactive oxygen species and disrupts the oxidative phosphorylation process (4). It may cause calcium overload, induce the opening of mitochondrial permeability transition pore and lead to mitochondrial dysfunction (22).

In the present study, we found that ADR reduced LVW/BW ratio, E/A ratio, SRe/SRa ratio, CVF, PVCA and the relative expression of Col I and Col III proteins, indicating that ADR can alleviate myocardial fibrosis and improve diastolic function in DCM rats. Until now there are no published articles discussing the effect of ADR on myocardial fibrosis and cardiac remodeling in DCM. However, ADR was proven to protect against liver fibrosis in a previous study. The authors measured the serum ADR levels, liver injury and oxidative stress in diet-induced non-alcoholic steatohepatitis mice. ADR knockout mice and palmitate treated primary hepatic cells were used to investigate the influence of ADR on liver injury. The results indicated that the treatment with ADR alleviated hepatocyte injury by upregulating the expression of Gclc, Gclm, and Gpx1 in a manner dependent on Nrf2 transcriptional activity and by increasing the glutathione levels (23). Therefore, the present study is the first article to report that ADR may reduce myocardial fibrosis in DCM rats. Moreover, based on the previous related researches on other disease models (12, 23), ADR seems to be effective in improving organ injury and fibrosis.

Moreover, our study also explored the role of mitochondrial dynamics in the process of ADR protecting against myocardial fibrosis in DCM rats. Mitochondria is extremely important for energy supply of heart. As we know, the heart requires large amounts of high-energy phosphates and accounts for ~8% of the total adenosine triphosphate consumption of the body. The majority of adenosine triphosphate is regenerated in the mitochondria via oxidative phosphorylation (5, 24). Mitochondria are highly dynamic organelles undergoing coordinated cycles of fission and fusion, which referred as mitochondrial dynamics (25). In mammalian cells, the primary regulators of mitochondrial fusion are dynamin-related mitofusins (mitofusin-1 and mitofusin-2) and optic atrophy protein 1 (OPA1). On the other side, mitochondrial fission 1 protein and the dynamin-related protein 1 (DRP1) are involved in mitochondrial fission. The balance of mitochondrial fusion and fission has been recognized as critical processes in the health of mitochondria and cells (5, 26).

The results of this study found that the balance of mitochondrial fusion and fission has been broken in DCM rats compared with CON rats via detecting mitochondrial dynamics related proteins. Besides, ADR can upregulate the relative expression of Mitofusin-1 and Mitofusin-2 proteins and downregulate the expression of Drp1 protein in DCM rats. Thapa et al. (27, 28) had also conducted a series of studies concerning the effect of ADR in cardiac cells and pre-diabetic obese mice. *In vitro* experiments, the stimulation of cultured cardiac cells with ADR may lead to decreased expression of the pyruvate dehydrogenase negative regulator PDK4, which reduced inhibitory PDH phosphorylation. *In vivo* study, cardiac glucose oxidation reduced in high fat diet animals. Acute ADR treatment increased cardiac glucose oxidation. ADR can modulate the expression of the mitochondrial acetyltransferase enzyme GCN5L1, altering the activity of fuel metabolism enzymes to favor glucose utilization. These findings suggest that ADR may be a key regulator of fuel substrate utilization in the heart and may provide a future therapeutic avenue in the treatment of DCM.

There are some limitations in this study. Firstly, much of the current knowledge regarding DCM is the result of studies performed using adult streptozotocin-induced diabetic rodents. However, the mechanism of DCM in this model may be related the direct actions of streptozotocin on muscle fibers independent of the diabetic phenotype. Therefore, future studies should be repeated in alternative models of DCM, such as Ins2 (+/Akita) mouse and high fat diet-fed models. Secondly, this is a preliminary study on the effect of ADR in DCM rats. Even though several mitochondrial dynamics related proteins had been detected, other signaling pathways or parameters related to energy metabolism and mitochondrial respiration have not been tested. Besides, this study did not report the results of *in vitro* experiments. The result mainly implied the phenomenon. Further study should be conducted to observe the effects of ADR on different specific cells in myocardial tissue. Moreover, this study did not explore the involvement of mitochondrial apoptosis in DCM. Therefore, further studies are needed in the future to confirm the mechanism of ADR in DCM.

## Conclusions

In summary, the present study demonstrated ADR could alleviate myocardial fibrosis and improve diastolic function in DCM rats. Our present study is the first to describe the effect of ADR on myocardial fibrosis in DCM rats. ADR may be a putative candidate for the treatment of DCM.

## Data Availability Statement

The raw data supporting the conclusions of this article will be made available by the authors, without undue reservation.

## Ethics Statement

The animal study was reviewed and approved by Ethics Committee of North Sichuan Medical College.

## Author Contributions

ML and JA: conception and design. ML: administrative support. ZS and JA: provision of study materials or patients. ZS, JA, and KT: collection and assembly of data. JA, KT, ZL, and YH: data analysis and interpretation. ML, JA, and YH: manuscript writing. All authors contributed to the article and approved the submitted version.

## Conflict of Interest

The authors declare that the research was conducted in the absence of any commercial or financial relationships that could be construed as a potential conflict of interest.
